# Four new corticioid species in Trechisporales (Basidiomycota) from East Asia and notes on phylogeny of the order

**DOI:** 10.3897/mycokeys.48.31956

**Published:** 2019-03-08

**Authors:** Shi-Liang Liu, Hai-Xia Ma, Shuang-Hui He, Yu-Cheng Dai

**Affiliations:** 1 Institute of Microbiology, Beijing Forestry University, Beijing 100083, China Beijing Forestry University Beijing China; 2 Institute of Tropical Bioscience and Biotechnology, Chinese Academy of Tropical Agricultural Sciences, Hainan Key Laboratory of Tropical Microbe Resources, Haikou 571101, China Institute of Tropical Bioscience and Biotechnology, Chinese Academy of Tropical Agricultural Sciences Haikou China

**Keywords:** Hydnodontaceae, *Sistotremastrum* family, phylogeny, taxonomy, wood-inhabiting fungi

## Abstract

Four new species in Trechisporales from East Asia, *Dextrinocystiscalamicola*, *Subulicystidiumacerosum*, *S.tropicum* and *Tubuliciumbambusicola*, are described and illustrated, based on morphological and molecular evidence. The phylogeny of Trechisporales was inferred from a combined dataset of ITS-nrLSU sequences. In the phylogenetic tree, *Sistotremastrum* formed a family-level clade of its own, sister to the Hydnodontaceae clade formed by all other genera. *Dextrinocystis*, is for the first time, confirmed as a member of Hydnodontaceae. A key to all the accepted genera in Trechisporales is given.

## Introduction

**Figure 1.**
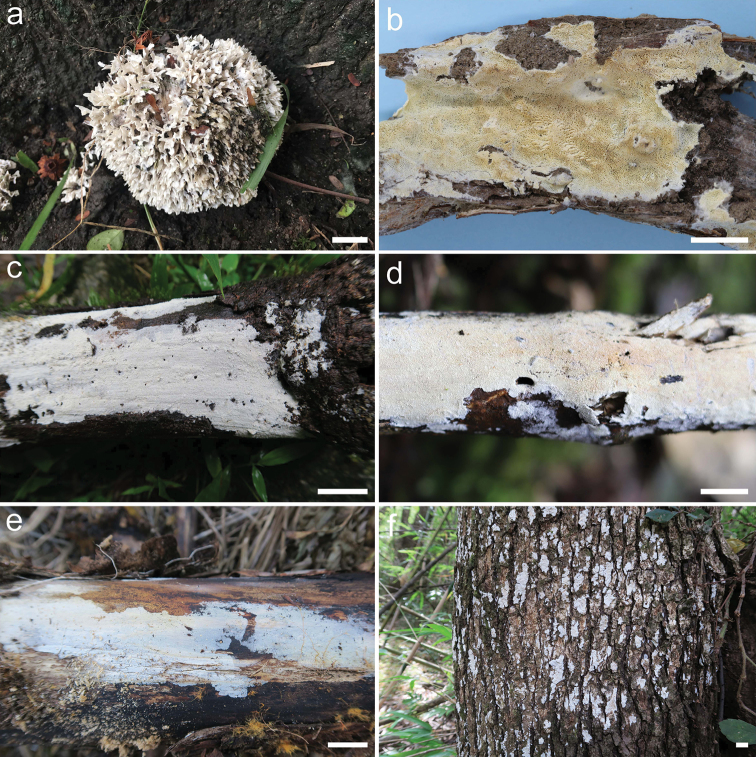
Basidiomata of Trechisporales. **a***Scytinopogonpallescens* (Bres.) Singer (He 5192) **b***Porpomycessubmucidus* F. Wu & C.L. Zhao (Dai 13708) **c***Fibrodontiaalba* Yurchenko & Sheng H. Wu (He 4761) **d***Trechispora* sp. (He 5491) **e***Subulicystidium* sp. (He 3048) **f***Tubuliciumraphidisporum* (Boidin & Gilles) Oberw., Kisim.-Hor. & L.D. Gómez (He 3191). Scale bar: 1 cm.

Trechisporales K.H. Larss. is a rather small but strongly supported order in Agaricomycotina (Hibbett et al. 2007; Larsson 2007). At present, eight to twelve genera, *Brevicellicium* K.H. Larss. & Hjortstam, *Fibriciellum* J. Erikss. & Ryvarden, *Fibrodontia*Parmasto, *Luellia* K.H. Larss. & Hjortstam, *Porpomyces* Jülich, *Subulicystidium* Parmasto, *Trechispora* P. Karst. (type genus, including *Cristelloporia* I. Johans. & Ryvarden, *Echinotrema* Park-Rhodes, *Hydnodon* Banker and *Scytinopogon* Singer) and *Tubulicium* Oberw., are placed in the family Hydnodontaceae, while *Sistotremastrum* J. Erikss. should be placed in a family of its own (Larsson 2001, 2007; Larsson et al. 2004; Binder et al. 2005; Hibbett et al. 2007, 2014; Birkebak et al. 2013; Telleria et al. 2013a). In addition, four genera, *Dextrinocystis* Gilb. & M. Blackw., *Dextrinodontia* Hjortstam & Ryvarden, *Brevicellopsis* Hjortstam & Ryvarden and *Litschauerella* Oberw. were listed as possible candidates of Hydnodontaceae waiting for molecular confirmation (Larsson 2007; Hibbett et al. 2014). Except for *Scytinopogon* and *Trechisporathelephora* (Lév.) Ryvarden, all the taxa in Trechisporales have resupinate basidiomata and most of them have a non-poroid hymenophore (Fig. 1, Albee-Scott and Kropp 2011; Hibbett et al. 2014). However, the microscopic characters vary significantly amongst different genera and some of them were surprisingly placed in the order solely based on molecular phylogeny (Larsson 2007; Bernicchia and Gorjón 2010).

Except for *Trechispora*, the largest genus in the order, most genera in Trechisporales have mostly few species and some are still monotypic. However, in recent years, many new species have been described, based on both DNA sequence data and morphological characters. Wu et al. (2015) described a cryptic species of *Porpomycesmucidus* (Pers.) Jülich, based mainly on sequence data. Ordynets et al. (2018) studied the short-spored species of *Subulicystidium* and recognised eleven new species. Tens specimens of Trechisporales were collected from East Asia by the senior authors in the past three years. The purposes of the present paper are to study these specimens by using morphological and molecular methods and discuss the phylogeny of the Trechisporales, based on expanded sampling.

## Materials and methods

### Morphological studies

Voucher specimens were deposited in the herbaria of Beijing Forestry University, Beijing, China (BJFC) and in the Centre for Forest Mycology Research, U.S. Forest Service, Madison, USA (CFMR). Freehand sections were made from dried basidiomata and mounted in 0.2% cotton blue in lactic acid, 1% phloxine (w/v) or Melzer’s reagent. Microscopic examinations were carried out with a Nikon Eclipse 80i microscope (Nikon Corporation, Japan) at magnifications up to 1000×. Drawings were made with the aid of a drawing tube. All measurements were carried out with sections mounted in Melzer’s reagent. The following abbreviations are used: L = mean spore length, W = mean spore width, Q = L/W ratio, n (a/b) = number of spores (a) measured from given number of specimens (b). Colour names and codes follow Kornerup and Wanscher (1978).

### DNA extraction and sequencing

The CTAB plant genome rapid extraction kit DN14 (Aidlab Biotechnologies Co. Ltd, Beijing) was used for DNA extraction and PCR amplification from dried specimens. The ITS1-5.8S-ITS2 and partial nrLSU markers were amplified with the primer pairs ITS5/ITS4 (White et al. 1990) and LR0R/LR7 (Vilgalys and Hester 1990). The PCR procedures followed Liu et al. (2017). DNA sequencing was performed at Beijing Genomics Institute and the sequences were deposited in GenBank (Benson et al. 2018). The sequence quality control followed Nilsson et al. (2012). BioEdit v.7.0.5.3 (Hall 1999) and Geneious v.11.1.15 (Kearse et al. 2012) were used for chromatogram check and contig assembly.

### Phylogenetic analyses

The molecular phylogeny was inferred from a combined dataset of ITS1-5.8S-ITS2-nrLSU sequences of Trechisporales sensu Larsson (2007) (Table 1). *Hyphodontiafloccosa* (Bourdot & Galzin) J. Erikss. and *H.subalutacea* (P. Karst.) J. Erikss. were selected as the outgroup (Wu et al. 2015). The sequences of ITS and nrLSU were aligned separately using MAFFT v.7 (Katoh et al. 2017, http://mafft.cbrc.jp/alignment/server/) with the G-INS-i iterative refinement algorithm. The separate alignments were concatenated using Mesquite v.3.5.1 (Maddison and Maddison 2018). The combined alignments were deposited in TreeBase (http://treebase.org/treebase-web/home.html, submission ID: 23620).

**Table 1.** Species and sequences used in the phylogenetic analyses.

Taxa	Voucher	Locality	ITS	nrLSU	Reference
* Brevicellicium exile *	MA-Fungi 26554	Spain	HE963777	HE963778	Telleria et al. (2013a)
* B. olivascens *	MA-Fungi 41366	Spain	HE963785	HE963786	Telleria et al. (2013a)
*B.* sp	MPM 2012	Portugal	–	HE963774	Telleria et al. (2013a)
* Dextrinocystis calamicola *	BJFC: He 5693	China	MK204533	MK204546	This study
* D. calamicola *	BJFC: He 5700	China	MK204534	MK204547	This study
	BJFC: He 5701	China	–	MK204548	This study
* Fibrodontia alba *	TNM: F25503	Taiwan	JQ612713	JQ612714	Yurchenko and Wu (2014)
* F. alba *	BJFC: He 4761	China	MK204529	MK204541	This study
* F. brevidens *	TNM: Wu 9807-16	Taiwan	KC928276	KC928277	Yurchenko and Wu (2014)
	BJFC: He 3559	China	MK204528	–	This study
* F. gossypina *	AFTOL-ID 599	–	DQ249274	AY646100	Unpublished
* Hyphodontia floccosa *	GB: Berglund 150-02	Sweden	DQ873618	DQ873617	Larsson et al. (2006)
* H. subalutacea *	GEL 2196	–	DQ340341	DQ340362	Unpublished
*Litschauerella* sp.	BJFC: He 3171	China	MK204555	MK204556	This study
* Porpomyces mucidus *	BJFC: Dai 12692	Czech Republic	KT157833	KT157838	Wu et al. (2015)
* P. submucidus *	BJFC: Cui 5183	China	KT152143	KT152145	Wu et al. (2015)
* Subulicystidium boidinii *	KAS: L 1584a	Reunion	MH041527	–	Ordynets et al. (2018)
* S. acerosum *	BJFC: He 3804	China	MK204539	MK204543	This study
* S. brachysporum *	O: F: KHL 16100	Brazil	MH000599	MH000599	Ordynets et al. (2018)
	BJFC: He 2207	USA	MK204532	MK204549	This study
* S. fusisporum *	GB: KHL 10360	Puerto Rico	MH041535	MH041567	Ordynets et al. (2018)
* S. grandisporum *	O: F: 506781	Costa Rica	MH041547	MH041592	Ordynets et al. (2018)
* S. harpagum *	KAS: L 1726a	Reunion	MH041532	MH041588	Ordynets et al. (2018)
* S. inornatum *	GB: KHL 10444	Puerto Rico	MH041558	MH041569	Ordynets et al. (2018)
* S. longisporum *	GB: KHL 14229	Sweden	MH000601	MH000601	Ordynets et al. (2018)
	BJFC: He 2981	China	–	MK204550	This study
* S. meridense *	GB: Hjm 16400	Brazil	MH041538	MH041604	Ordynets et al. (2018)
* S. nikau *	KAS: L 1296	Reunion	MH041513	MH041565	Ordynets et al. (2018)
* S. obtusisporum *	FR: Piepenbrink & Lotz-Winter W213-3-I	Germany	MH041521	MH041566	Ordynets et al. (2018)
* S. parvisporum *	KAS: L 0140	Reunion	MH041529	MH041590	Ordynets et al. (2018)
* S. perlongisporum *	TU 124388	Italy	UDB028355	UDB028355	Kõljalg et al. (2013)
	GB: KHL 16062	Brazil	MH000600	MH000600	Ordynets et al. (2018)
* S. rarocrystallinum *	O: F: 918488	Colombia	MH041512	MH041564	Ordynets et al. (2018)
* S. robustius *	GB: KHL 10813	Jamaica	MH041514	MH041608	Ordynets et al. (2018)
* S. tedersooi *	TU 110894	Vietnam	UDB014161	–	Kõljalg et al. (2013)
* S. tropicum *	BJFC: He 3968	China	MK204531	MK204544	This study
	BJFC: He 3583	China	MK204530	MK204542	This study
* Scytinopogon angulisporus *	TFB 13611	USA	–	JQ684661	Unpublished
* S. havencampii *	SFSU: DED 8300	Príncipe island	KT253946	KT253947	Desjardin and Perry (2015)
* S. pallescens *	BJFC: He 5192	Vietnam	–	MK204553	This study
* Sistotremastrum guttuliferum *	MA-Fungi 82105	Portugal	JX310445	–	Telleria et al. (2013b)
* S. guttuliferum *	BJFC: He 3338	China	MK204540	MK204552	This study
* S. niveocremeum *	CBS 427.54	France	MH857380	MH868920	Vu et al. (2019)
* S. suecicum *	GB: KHL11849	Sweden	EU118666	EU118667	Larsson (2007)
* Trechispora alnicola *	AFTOL-ID 665	–	–	AY635768	Unpublished
* T. araneosa *	GB: KHL 8570	Sweden	AF347084	AF347084	Larsson et al. (2004)
* T. bispora *	CBS 142.63	Australia	MH858241	MH869842	Vu et al. (2019)
* T. confinis *	GB: KHL 11064	Sweden	AF347081	AF347081	Larsson et al. (2004)
* T. farinacea *	TUB 011825	Germany	EU909231	EU909231	Krause et al. (2011)
* T. hymenocystis *	GB: KHL 8795	Sweden	AF347090	AF347090	Larsson et al. (2004)
* T. kavinioides *	GB: KGN 981002	Norway	AF347086	AF347086	Larsson et al. (2004)
* T. mollusca *	CBS 439.48	Canada	MH856428	–	Vu et al. (2019)
* T. nivea *	GB: G. Kristiansen	Norway	–	AY586720	Larsson et al. (2004)
* Tubulicium bambusicola *	BJFC: He 4776	China	MK204536	MK204551	This study
* T. bambusicola *	BJFC: He 4058	Thailand	MK204535	–	This study
* T. raphidisporum *	BJFC: He 2851	China	MK204538	MK204554	This study
	BJFC: He 3191	China	MK204537	MK204545	This study
* T. vermiculare *	GEL 5015	–	AJ406424	–	Langer (2002)
* T. vermiferum *	GB: KHL 8714	Norway	–	AY463477	Larsson et al. (2004)

For both Maximum Likelihood (ML) and Bayesian Inference (BI), a partitioned analysis was performed with the following four partitions: ITS1, 5.8S, ITS2 and nrLSU. The ML analysis was performed using RAxML v.8.2.10 (Stamatakis 2014) with the bootstrap values (ML-BS) obtained from 1,000 replicates and the GTRGAMMA model of nucleotide evolution. The BI was performed using MrBayes 3.2.6 (Ronquist et al. 2012). The best-fit substitution model for each partitioned locus was estimated separately with jModeltest v.2.17 (Darriba et al. 2012) by restricting the search to models that can be implemented in MrBayes. Two runs of four Markov chains were run for 4,000,000 generations until the split deviation frequency value was lower than 0.01. The convergence of the runs was checked using Tracer v.1.7 (Rambaut et al. 2018). Trees and model parameters were sampled every 100^th^ generation. The first quarter of the trees, which represented the burn-in phase of the analyses, was discarded and the remaining trees were used to build a majority rule consensus tree and to calculate Bayesian posterior probabilities (BPP). All trees were visualised in FigTree 1.4.2 (Rambaut 2014).

## Results

### Phylogenetic inference

**Figure 2.**
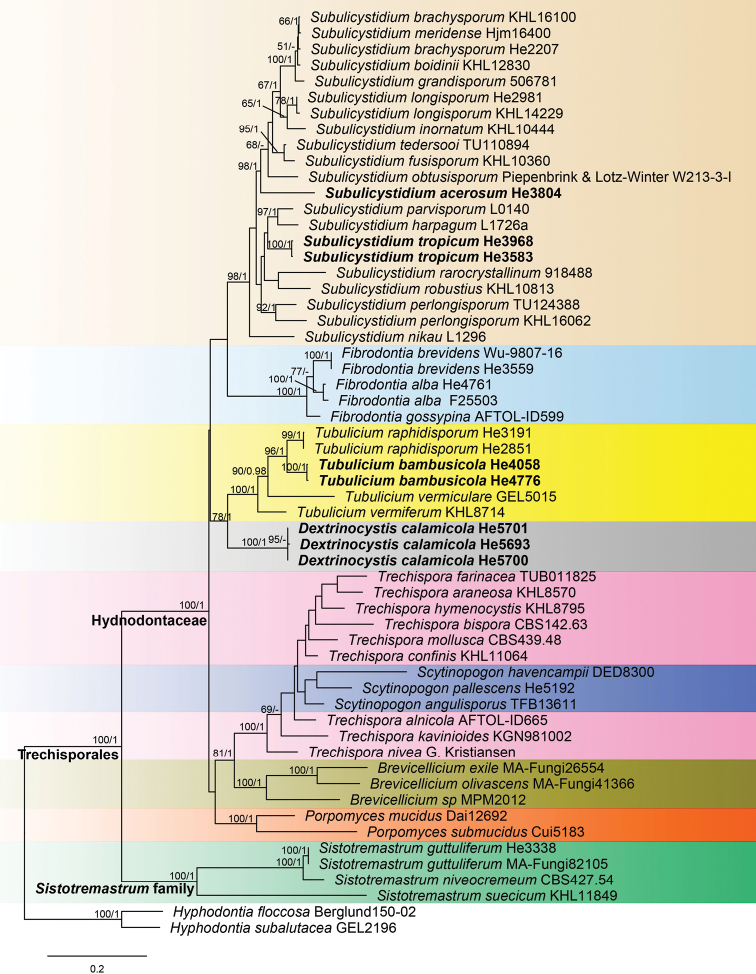
Phylogeny of Trechisporales inferred from ITS-nrLSU sequences. Topology is from ML analysis with maximum likelihood bootstrap support values (≥ 50, former) and Bayesian posterior probability values (≥ 0.95, latter) shown along the branches. Different genera are indicated as coloured blocks. The new species are set in bold. Scale bar: 0.2 nucleotide substitutions per site.

The ITS-nrLSU sequence dataset contained 50 ITS and 51 nrLSU sequences from 58 samples representing 45 ingroup taxa and the outgroup (Table 1). Fourteen ITS and 15 nrLSU sequences were generated for this study. jModelTest suggested GTR+G, SYM+I+G, GTR+I+G and GTR+I+G to be the best-fit models of nucleotide evolution for ITS1, 5.8S, ITS2 and nrLSU markers, respectively, for the Bayesian analysis. BI analysis resulted in an almost identical tree topology compared to the ML analysis and no significant conflicts were found between the two analyses. Only the ML tree is shown in Fig. 2 with ML bootstrap values ≥ 50% and Bayesian posterior probabilities ≥ 0.95 labelled along the branches.

In the tree (Fig. 2), two large clades, corresponding to Hydnodontaceae and *Sistotremastrum* family, were strongly supported. Except for *Sistotremastrum*, the other eight genera sampled were nested within the Hydnodontaceae clade. The genera *Brevicellicium*, *Fibrodontia*, *Porpomyces* and *Subulicystidium* were strongly supported as monophyletic lineages. *Dextrinocystiscalamicola*, the first species sequenced in the genus, formed a sister lineage to *Tubulicium* with relatively strong support (ML-BS = 78%, BPP = 1). The three species of *Scytinopogon* were nested within the *Trechispora* lineage. *Subulicystidiumacerosum* and *S.tropicum* formed distinct lineages in the genus, while *Tubuliciumbambusicola* is closely related to *T.raphidisporum*.

### Taxonomy

#### Dextrinocystis calamicola

Taxon classificationFungiTrechisporalesHydnodontaceae

S.H. He & S.L. Liu sp. nov.

828718

Fig. 3

##### Typification.

CHINA. Fujian Province, Wuyishan County, Wuyishan Nature Reserve, on dead culms of *Calamus*, 3 Oct 2018, He 5701 (holotype, BJFC 026763).

##### Etymology.

“*calamicola*” refers to growing on *Calamus*.

##### Basidiomata.

Annual, resupinate, effused, thin, soft, easily separated from the substrate, at first as irregular small patches, later confluent up to 15 cm long, 2 cm wide. Hymenophore surface smooth, orange white (5A2) to greyish-orange [5B(3–5)], finely cracked with age; margin thinning out, fimbriate, slightly paler than hymenophore surface, becoming indistinct with age.

##### Microscopic structures.

Hyphal system monomitic; generative hyphae with clamp connections, hyaline, thin-walled, frequently branched and septate, loosely interwoven, 2–3 µm in diam. Cystidia-like branches present, branched from subicular hyphae, embedded, hyaline, thick-walled, encrusted at apex, 20–30 × 1.5–2 µm. Hymenial cystidia abundant, subulate, projecting beyond hymenium, bi- or multi-rooted, hyaline, distinctly thick-walled with a narrow lumen, slightly encrusted at apex, distinctly dextrinoid, 50–110 × 5–6 µm. Basidia suburniform to subclavate, hyaline, thin-walled, with 4 sterigmata and a basal clamp connection, 20–30 × 5–8 µm; sterigmata mostly cylindrical with a blunt tip; basidioles in shape similar to basidia, but slightly smaller. Basidiospores abundant, oblong ellipsoid to short cylindrical, hyaline, thin-walled, smooth, negative in Melzer’s reagent, acyanophilous, (7–)7.5–8.8(–9) × (3.2–)3.3–4 µm, L = 8.1 µm, W = 3.7 µm, Q = 2.1–2.2 (n = 60/2).

##### Additional specimens examined.

CHINA. Fujian Province, Wuyishan County, Wuyishan Nature Reserve, on dead culms of *Calamus*, 3 Oct 2018, He 5693 (BJFC 026755) & He 5700 (BJFC 026762).

##### Remarks.

The thin whitish basidiomata on a palm tree, distinctly thick-walled cystidia with a dextrinoid reaction in Melzer’s reagent, presence of small cystidia-like branches and short cylindrical basidiospores indicate that the new species is a member of *Dextrinocystis*. Two species, *D.capitata* (D.P. Rogers & Boquiren) Gilb. & M. Blackw. and *D.macrospora* (Liberta) Nakasone have been reported in the genus, both of which differ from *D.calamicola* by having much larger basidiospores (11–14 × 3–4 µm for *D.capitata* in Gilbertson and Blackwell 1988; 12–19 × 4.5–7 µm for *D.macrospora* in Liberta 1960) and a distribution in America. In the phylogenetic tree, *D.calamicola* formed a sister lineage to *Tubulicium* with relatively strong support (Fig. 2).

**Figure 3.**
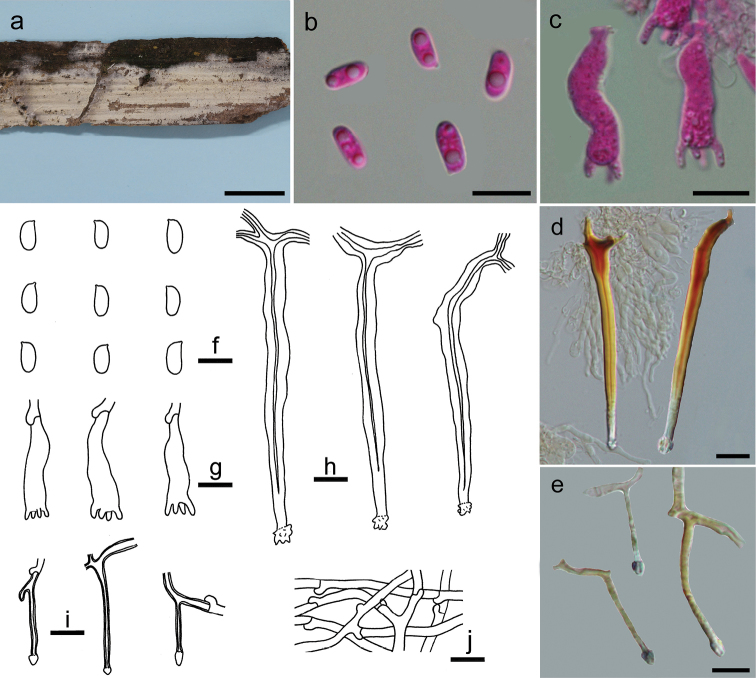
*Dextrinocystiscalamicola* (holotype, He 5701). **a** basidiomata **b, f** basidiospores **c, g** basidia **d, h** cystidia **e, i** cystidia-like branches in subiculum **j** subicular hyphae. Scale bars: 1 cm (**a**), 10 µm (**b–j**). **b, c** Taken in phloxine **d, e** taken in Melzer’s reagent.

#### Subulicystidium acerosum

Taxon classificationFungiTrechisporalesHydnodontaceae

S.H. He & S.L. Liu sp. nov.

828719

Fig. 4

##### Typification.

CHINA. Guizhou Province, Libo County, Maolan Nature Reserve, on fallen angiosperm trunk, 16 Jun 2016, He 3804 (holotype, BJFC 022303).

##### Etymology.

“*acerosum*” refers to the presence of numerous needle-like crystals.

##### Basidiomata.

Annual, resupinate, effused, very thin, easily separated from the substrate, up to 6 cm long, 2 cm wide. Hymenophore surface smooth, more or less arachnoid, white (5A1) to orange grey (5B2); margin undifferentiated.

##### Microscopic structures.

Hyphal system monomitic; generative hyphae with clamp connections, hyaline, thin-walled, frequently branched and septate, loosely interwoven, 2–3.5 µm in diam. Cystidia abundant, subulate, projecting beyond hymenium, hyaline, thick-walled and regularly covered with rectangular crystals at basal part, thin-walled and smooth at apex part, 50–100 × 3–5 µm. Crystals numerous, distributed in whole section or more commonly attached on cystidia, acerose, hyaline. Basidia short clavate, hyaline, thin-walled, with 4 sterigmata and a basal clamp connection, 15–20 × 4–5.5 µm; basidioles in shape similar to basidia, but slightly smaller. Basidiospores narrowly fusiform to slightly vermicular, hyaline, thin-walled, smooth, negative in Melzer’s reagent, acyanophilous, (14.5–)15.5–18(–20) × 1.8–2.2 µm, L = 16.6 µm, W = 2 µm, Q = 8.3 (n = 30/1).

##### Remarks.

*Subulicystidiumacerosum* is characterised by the long and narrow basidiospores and presence of numerous acerose crystals. The species is similar to *S.longisporum* (Pat.) Parmasto, which differs in having slightly shorter and wider basidiospores (12–16 × 2–3 µm, Q < 7, Ordynets et al. 2018). *Subulicystidiumcochleum* Punugu is similar to *S.acerosum* by sharing needle-like crystals but differs in having larger basidiospores (20–27 × 2–3 µm, Punugu et al. 1980; Ordynets et al. 2018). Phylogenetically, *S.acerosum* is distinct from all the other sampled species of *Subulicystidium* (Fig. 2).

**Figure 4.**
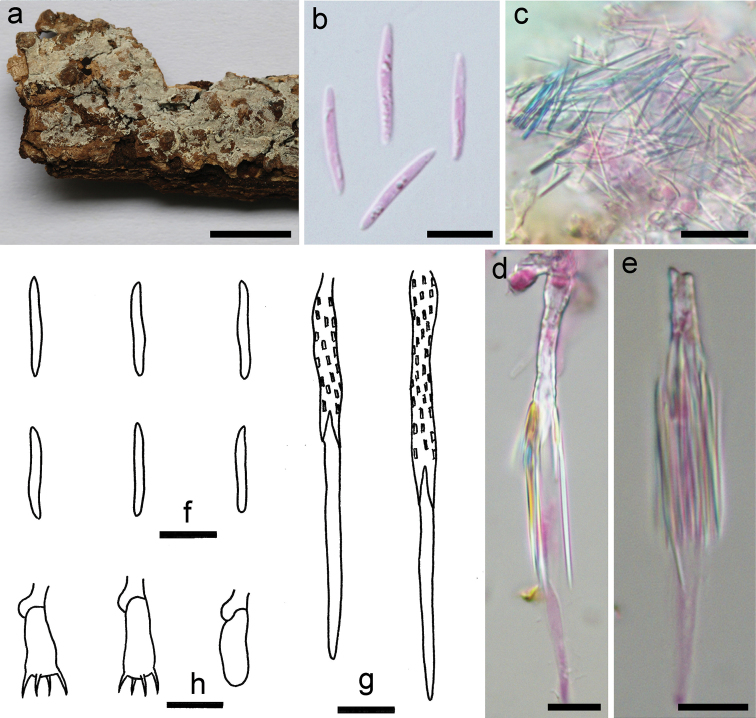
*Subulicystidiumacerosum* (holotype, He 3804). **a** basidiomata **b, f** basidiospores **c** acerose crystals **d, e, g** cystidia **h** basidia and a basidiole. Scale bars: 1 cm (**a**), 10 µm (**b–h**). **b–e** Taken in phloxine.

#### Subulicystidium tropicum

Taxon classificationFungiTrechisporalesHydnodontaceae

S.H. He & S.L. Liu sp. nov.

828720

Fig. 5

##### Typification.

CHINA. Hainan Province, Wuzhishan County, Wuzhishan Nature Reserve, on fallen angiosperm branch, 10 Jun 2016, He 3968 (holotype, BJFC 022470).

##### Etymology.

“*tropicum*” refers to the distribution in tropical areas.

##### Basidiomata.

Annual, resupinate, effused, very thin, separable from the substrate, up to 10 cm long, 3 cm wide. Hymenophore surface smooth, white (5A1), orange grey (5B2) to greyish-orange [5B(3–4)], not cracked; margin undifferentiated.

##### Microscopic structures.

Hyphal system monomitic; generative hyphae with clamp connections, hyaline, slightly thick-walled, frequently branched and septate, loosely interwoven, 2–3.5 µm in diam. Cystidia abundant, subulate, projecting beyond hymenium, hyaline, thick-walled and regularly covered with rectangular crystals except at the apex, 40–70 × 3–5 µm. Basidia subclavate to suburniform, hyaline, thin-walled, with 4 sterigmata and a basal clamp connection, 12–17 × 4–5 µm; basidioles in shape similar to basidia, but slightly smaller. Basidiospores fusiform to slightly vermicular, hyaline, thin-walled, smooth, negative in Melzer’s reagent, acyanophilous, 11–12.5(–13) × 1.8–2.2 µm, L = 11.9 µm, W = 2 µm, Q = 5.95 (n = 30/1).

##### Additional specimens examined.

CHINA. Hainan Province, Baoting County, Qixianling Forest Park, on fallen angiosperm branch, 18 Mar 2016, He 3583 (BJFC 022083).

##### Remarks.

*Subulicystidiumtropicum* resembles *S.acerosum* and *S.perlongisporum* Boidin & Gilles by sharing narrow basidiospores in the genus, but differs from *S.acerosum* in having shorter basidiospores and lacking the needle-like crystals and from *S.perlongisporum* in having much shorter basidiospores and a tropical distribution (16–25 × 1.5–2.5 µm for *S.perlongisporum* in Ordynets et al. 2018). The new species is also similar to *S.longisporum*, but differs in having slender basidiospores and a tropical distribution. In the phylogenetic tree, *S.tropicum* formed a distinct lineage in *Subulicystidium* (Fig. 2).

**Figure 5.**
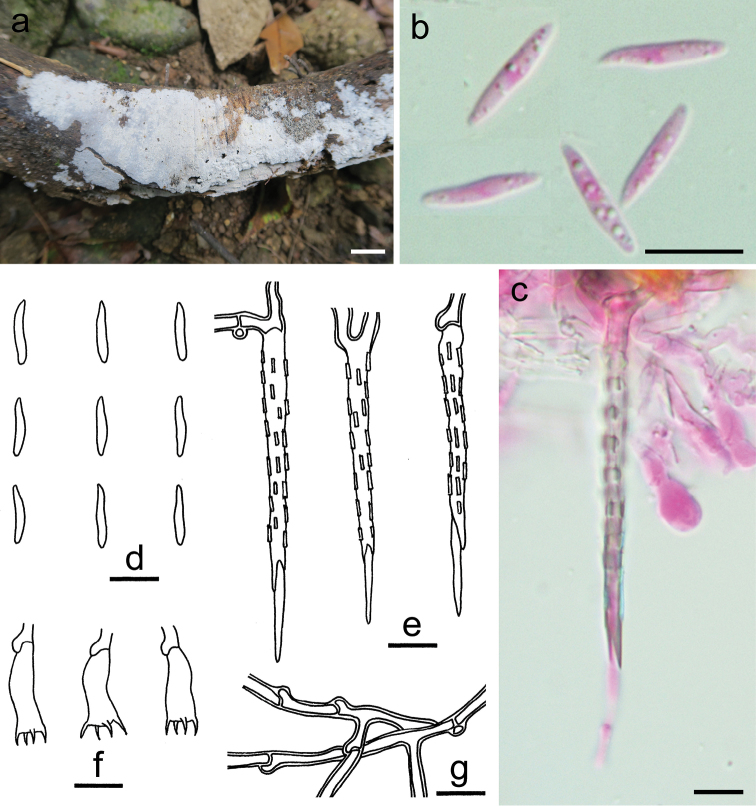
*Subulicystidiumtropicum* (holotype, He 3968). **a** basidiomata **b, d** basidiospores **c, e** cystidia **f** basidia **g** subicular hyphae. Scale bars: 1 cm (**a**), 10 µm (**b–g**). **b, c** Taken in phloxine.

#### Tubulicium bambusicola

Taxon classificationFungiTrechisporalesHydnodontaceae

S.H. He & S.L. Liu sp. nov.

828721

Fig. 6

##### Typification.

THAILAND. Chiang Rai Province, Doi Mae Salong, on dead culms of bamboo, 22 Jul 2016, He 4058 (holotype, BJFC 023499).

##### Etymology.

“*bambusicola*” refers to growing on bamboo.

##### Basidiomata.

Annual, resupinate, effused, closely adnate, thin, at first as irregular small patches, later confluent up to 15 cm long, 5 cm wide. Hymenophore surface smooth, pilose under lens due to the projecting cystidia, pale orange (5A3) to greyish-orange [5B(3–6)], finely cracked with age; margin undifferentiated.

##### Microscopic structures.

Hyphal system monomitic; generative hyphae with clamp connections, hyaline, thin-walled, moderately branched, frequently septate, loosely interwoven, 2–3 µm in diam. Cystidia abundant, subulate, projecting beyond hymenium, multi-rooted, hyaline, distinctly thick-walled, slightly amyloid, covered with dendroid branching hyphae, 70–100 × 10–16 µm. Basidia subclavate, hyaline, thin-walled, with 4 sterigmata and a basal clamp connection, 18–25 × 8–10 µm; basidioles in shape similar to basidia, but slightly smaller. Basidiospores narrowly fusiform to vermicular, bi-apiculate, hyaline, thin-walled, smooth, negative in Melzer’s reagent, acyanophilous, (17–)20–29(–30) × (2–)2.2–3(–3.2) µm, L = 23.9 µm, W = 2.6 µm, Q = 9–9.5 (n = 60/2).

##### Additional specimens examined.

CHINA. Guizhou Province, Libo County, Maolan Nature Reserve, on rotten culms of bamboo, 11 Jul 2017, He 4776 (BJFC 024293).

##### Remarks.

*Tubuliciumbambusicola* is distinguished by its large vermicular basidiospores and growing on bamboo. Three taxa, *T.raphidisporum* (Boidin & Gilles) Oberw., Kisim.-Hor. & L.D. Gómez, *T.vermiferum* (Bourdot) Oberw. and T.vermiferumvar.hexasterigmatum J. Kaur & Dhingra are similar to *T.bambusicola* by sharing long vermicular basidiospores but differ in the width of basidiospores (≥ 3.5 µm) and growing on woody plant. *Tubuliciumjunci-acuti* Boidin & Gaignon on *Juncusacutus* differs from *T.bambusicola* by having shorter and wider basidiospores (15–20 × 3–4.25 µm, Boidin and Gaignon 1992).

**Figure 6.**
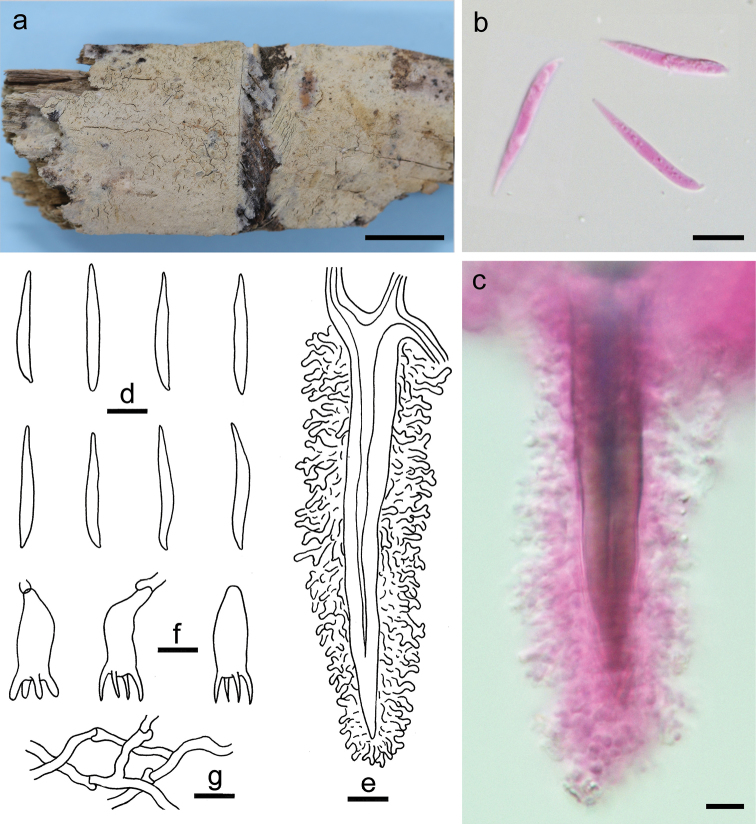
*Tubuliciumbambusicola* (holotype, He 4058). **a** basidiomata **b, d** basidiospores **c, e** cystidia **f** basidia and a basidiole **g** subicular hyphae. Scale bars: 1 cm (**a**), 10 µm (**b–g**). **b, c** Taken in phloxine.

## Discussion

Nine genera in the Trechisporales were included in the present analyses and the results mostly agree with previous studies (Larsson 2007; Birkebak et al. 2013; Telleria et al. 2013a). Most of the sampled genera were retrieved as monophyletic except *Scytinopogon*, which was nested within the *Trechispora* lineage (Fig. 2). A *Dextrinocystis* species was sequenced for the first time and its position in Hydnodontaceae was confirmed. As indicated by the morphology (Burdsall and Nakasone 1983; Gilbertson and Blackwell 1988; Moreno and Esteve-Raventós 2007; Nakasone 2013), the genus is closely related to *Tubulicium*. However, *Tubulicium* is morphologically heterogenous, with different basidiospores (Moreno and Esteve-Raventós 2007; Hjortstam and Ryvarden 2008) and only species with fusiform to vermicular basidiospores were sequenced. Moreover, *Dextrinocystis* is well distinguished from *Tubulicium* by its distinctly dextrinoid cystida and cylindrical basidiospores (Gilbertson and Blackwell 1988; Nakasone 2013). Thus, at present, the authors prefer to retain them as separate genera until more species are sequenced.

*Subulicystidium* is a well-circumscribed genus characterised by the unique cystidia encrusted with rectangular crystals and fusiform to vermicular basidiospores (Bernicchia and Gorjón 2010; Ordynets et al. 2018). Although all the sampled species formed a strongly supported lineage in the tree (Fig. 2), the species *S.oberwinkleri* Ordynets, Riebesehl & K.H.Larss. was not congeneric with other species and excluded from our analyses. Ordynets et al. (2018) showed that *S.oberwinkleri* formed a distinct basal lineage in the ITS-nrLSU tree. The phylogenetic position of the species in Trechisporales needs to be further studied.

### Key to accepted genera in Trechisporales

**Table:** 

1	Basidiomata clavarioid	*** Scytinopogon ***
–	Basidiomata resupinate or stipitate hydnoid	**2**
2	Hymenophore poroid	**3**
–	Hymenophore non-poroid	**4**
3	Basidiospores smooth	*** Porpomyces ***
–	Basidiospores ornamented	***Trechispora* p.p.**
4	Basidiomata brown	*** Luellia ***
–	Basidiomata light coloured	**5**
5	Cystidia present, large and distinct	**6**
–	Cystidia absent or indistinct	**8**
6	Cystidia distinctly dextrinoid in Melzer’s reagent	*** Dextrinocystis ***
–	Cystidia negative or amyloid in Melzer’s reagent	**7**
7	Cystidia regularly encrusted with rectangular crystals	*** Subulicystidium ***
–	Cystidia usually covered with dendroid hyphae	*** Tubulicium ***
8	Generative hyphae with ampullate septa	***Trechispora* p.p.**
–	Generative hyphae without ampullate septa	**9**
9	Subhymenial hyphae isodiametric	*** Brevicellicium ***
–	Subhymenial hyphae not isodiametric	**10**
10	Hyphal system dimitic; basidia with 4 sterigmata	*** Fibrodontia ***
–	Hyphal system monomitic; basidia with 4–8 sterigmata	*** Sistotremastrum ***

## Supplementary Material

XML Treatment for Dextrinocystis calamicola

XML Treatment for Subulicystidium acerosum

XML Treatment for Subulicystidium tropicum

XML Treatment for Tubulicium bambusicola
